# Crystal structures of three *N*-(3-acetyl­phen­yl)quinoline-2-carboxamides

**DOI:** 10.1107/S2056989017006272

**Published:** 2017-05-05

**Authors:** Diana Peña-Solórzano, Burkhard König, Cesar A. Sierra, Cristian Ochoa-Puentes

**Affiliations:** a Grupo de Investigación en Macromoléculas, Departamento de Química, Universidad, Nacional de Colombia-Sede Bogotá, Carrera 45 # 26-85, A.A. 5997, Bogotá, Colombia; b Institute of Organic Chemistry, University of Regensburg, 93040-Regensburg, Germany

**Keywords:** crystal structure, quinoline, carboxamide, aceto­phenone.

## Abstract

In the title compounds, *N*-(5-acetyl-2-methyl­phen­yl)quinoline-2-carboxamide [C_19_H_16_N_2_O_2_, (I)], *N*-(5-acetyl-2-bromo­phen­yl)quinoline-2-carboxamide [C_18_H_13_BrN_2_O_2_, (II)] and *N*-(5-acetyl-2-ethynylphen­yl)quinoline-2-carboxamide [C_20_H_14_N_2_O_2_, (III)], the quinoline ring system is essentially planar and forms a dihedral angles of 3.68 (5) (I), 5.59 (7) (II) and 1.87 (6)° (III) with the acetyl-substituted ring. The mol­ecular structures of (I) and (III) each feature an intra­molecular N—H⋯N hydrogen bond, forming an *S*(5) ring, while in (II) an intra­molecular bifurcated N—H⋯(N,Br) hydrogen bond forms two *S*(5) rings. In the crystals, weak C—H⋯O hydrogen bonds link mol­ecules of (I) into *C*(7) chains long [010], mol­ecules of (II) into chains of *R*_2_^2^(8) rings along [110] and mol­ecules of (III) into *C*(8) chains along [010]. In (I), there are no significant π–π stacking inter­actions under 4 Å, but in both (II) and (III), π–π inter­actions link the weak hydrogen-bonded chains into layers parallel to (001) [centroid–centroid disttances of 3.748 (1) Å in (II) and 3.577 (1), 3.784 (1) and 3.780 (1) Å in (III)].

## Abstract 2

The hydrogen-bonding inter­actions in the crystal structures of *N*-(5-acetyl-2-methyl­phen­yl)quinoline-2-carboxamide, *N*-(5-acetyl-2-bromo­phen­yl)quinoline-2-carboxamide and *N*-(5-acetyl-2-ethynylphen­yl)quinoline-2-carboxamide are described. The latter two compounds also exhibit π–π inter­actions.

## Chemical context   

Amino­aceto­phenones, quinolines and carboxamides have been reported to possess many inter­esting pharmacological activities and they are characteristic components of a large number of biologically active compounds. The wide spectrum of biological effects of these kind of compounds includes anti­microbial (Nawar & Hosny, 2000 ▸), anti­convulsant (Pandeya *et al.*, 1998 ▸), cytotoxic (Zhao *et al.*, 2005 ▸), anti-malarial (Egan *et al.*, 1994 ▸), anti­proliferative (Chen *et al.*, 2006 ▸), anti­tuberculosis/anti­mycobacterial (Gonec *et al.*, 2012 ▸) activities, radioligands (Matarrese *et al.*, 2001 ▸, Belloli *et al.*, 2004 ▸), calpain inhibitors (Nam *et al.*, 2008 ▸), TPSO ligand (Blair *et al.*, 2013 ▸) and pharmaceutical medicaments (Weidmann *et al.*, 2008 ▸), among others.

## Structural commentary   

**Figure 1:**
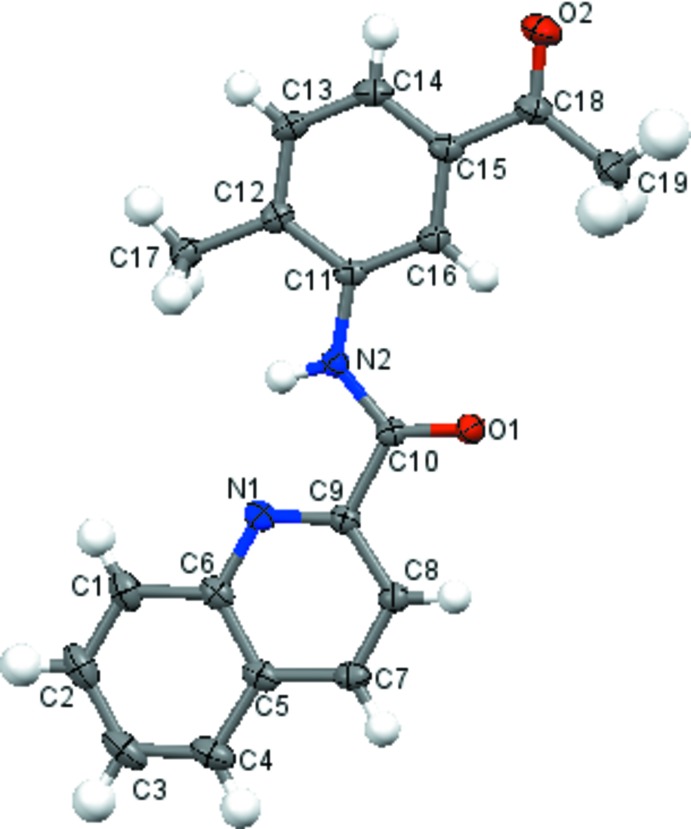
The mol­ecular structure of (I), with the atom-numbering scheme and displacement ellipsoids drawn at the 50% probability level.

The mol­ecular structure of title compounds (I), (II) and (III) are shown in Figs. 1 ▸, 2 ▸ and 3 ▸, respectively. The quinoline ring system [C1–C9/N1 in (I), C2–C10/N1 in (II) and C12–C20/N2 in (III)] in each compound is essentially planar with maximum deviations of 0.015 (1) Å for C3 in (I), 0.017 (2) Å for C3 in (II) and 0.013 (2) Å for C17 in (III). The quinoline ring system forms dihedral angles of 3.68 (5)° (I), 5.59 (7)° (II) and 1.87 (6)° (III) with the acetyl-substituted ring [C11–C16 in (I) and (II), C3–C8 in (III)]. In the mol­ecular structures of (I) and (III), an intra­molecular N—H⋯N hydrogen bond forms an *S*(5) ring while in (II) an intra­molecular bifurcated N—H⋯(N,Br) hydrogen bond forms two *S*(5) rings (Tables 1 ▸–3 ▸ ▸).
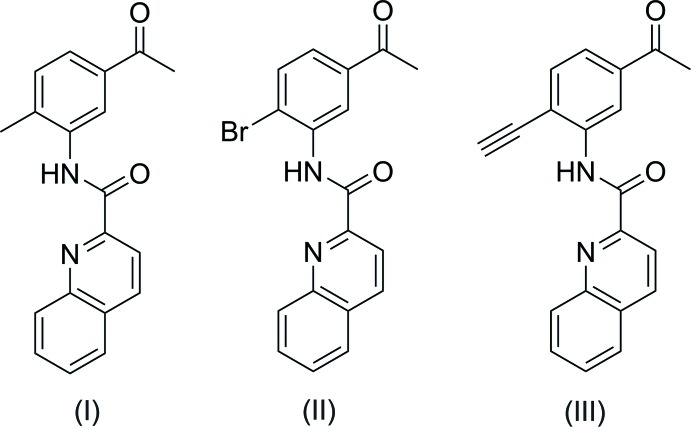


## Supra­molecular features   

**Figure 4:**
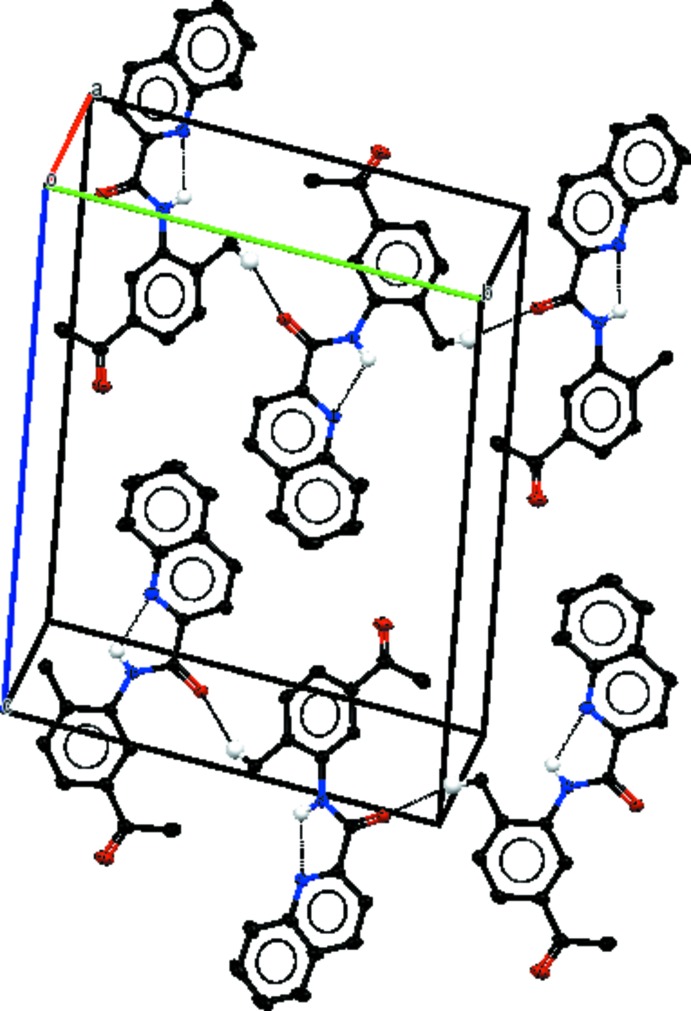
Part of the crystal structure of (I), with inter­molecular and intra­molecular hydrogen bonds shown as black dotted lines. Only H atoms involved in hydrogen bonds are shown.

**Figure 5:**
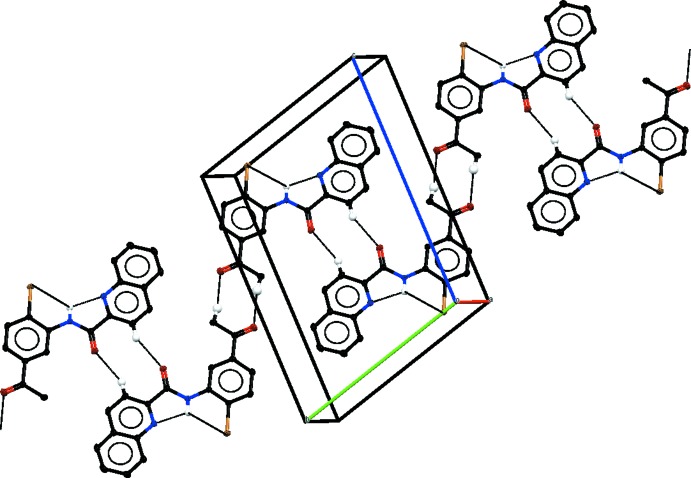
Part of the crystal structure of (II), with inter­molecular and intra­molecular hydrogen bonds shown as black dotted lines. Only H atoms involved in hydrogen bonds are shown.

**Figure 6:**
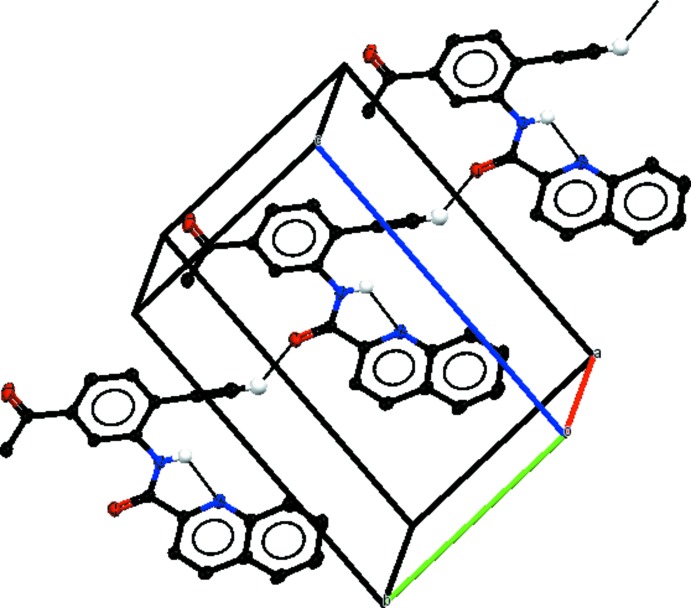
Part of the crystal structure of (III), with inter­molecular and intra­molecular hydrogen bonds shown as black dotted lines. Only H atoms involved in hydrogen bonds are shown.

In the crystals, weak C—H⋯O hydrogen bonds link mol­ecules of (I) into *C*(7) chains along [010] (Fig. 4 ▸), mol­ecules of (II) into chains of 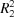
(8) rings along [110] (Fig. 5 ▸) and mol­ecules of (III) into *C*(8) chains along [010] (Fig. 6 ▸). In (I), there are no significant π–π stacking inter­actions under 4 Å but in (II) π–π inter­actions link the weak hydrogen-bonded chains into layers parallel to (001) [centroid–centroid distance *Cg*1⋯*Cg*2(1 + *x*, *y*, *z*) = 3.748 (1) Å; *Cg*1 and *Cg*2 are the centroids of the C5–C10 and N1/C2–C6 rings, respectively]. In (III), π–π inter­actions link the weak hydrogen-bonded chains into layers parallel to (001) with centroid–centroid distances *Cg*3⋯*Cg*4(−1 + *x*, −1 + *y*, −1 + *z*) = 3.577 (1), *Cg*4⋯*Cg*5(−*x* + 1, −*y* + 1, −*z* + 1) 3.784 (1) and *Cg*4⋯*Cg*5(−*x* + 2, −*y* + 1, −*z* + 1) = 3.780 (1) Å; *Cg*3, *Cg*4, and *Cg*5 are the centroids of the N2/C12–C16, C3–C8 and C15–C20 rings, respectively].

## Database survey   

A search of the Cambridge Structural Database (Groom *et al.* 2016 ▸; Version 1.18, April 2016) for similar compounds with an *N*-phenyl­quinoline-2-carboxamide skeleton resulted in twelve hits. One entry for the compound without substituents is reported (Jing & Qin, 2007 ▸). Eight are structures substituted in the 4-position of the phenyl group: one meth­oxy group (Qi *et al.*, 2003 ▸) and another a nitro group (Jing & Qin, 2008 ▸); one chlorine and one fluorine (Khavasi *et al.*, 2014 ▸), and two reports each for bromine (Bobal *et al.*, 2012 ▸; Khavasi *et al.*, 2014 ▸) and iodine (Qi *et al.*, 2003 ▸; Khavasi *et al.*, 2014 ▸). The rest have large organic substituents.

## Synthesis and crystallization   

**Figure 7:**
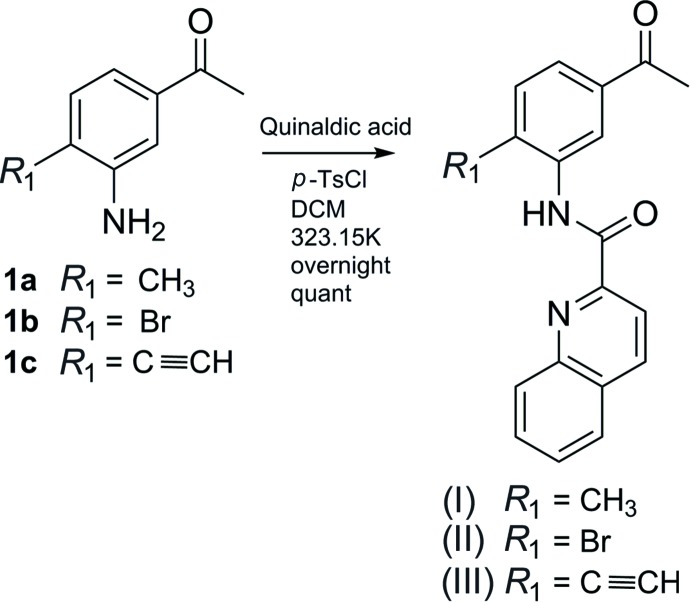
The reaction scheme for the synthesis of the title compounds

Compounds (I)–(III) were prepared by refluxing a mixture of quinaldic acid, tri­ethyl­amine, *p*-toluene­sulfonyl chloride and the corresponding substituted amino­aceto­phenones (**1a**–**c**) for 24 h in DCM (Fig. 7 ▸). Acetic acid 5% was added to quench the reaction, and the organic phase was washed three times with water. After evaporation of DCM, the compounds were purified by silica column chromatography (penta­ne:ethyl acetate 2:1). Single crystals were obtained by slow evaporation of the respective solutions of the compounds in di­chloro­methane into a closed flask with petroleum ether.

***N*** **-(5-acetyl-2-methyl­phen­yl)quinoline-2-carboxamide (I):** Light-yellow solid (0.700 g, yield quant, *R*_f_ PE/EA 2:1 0.52). **^1^H NMR (400 MHz, CDCl_3_): δ** 8.95 (*d*, *^3^J* = 7.7 Hz, 1H, quinol), 8.40 (*s*, 2H, ArH quinol), 8.17 (*d*, *^3^J* = 8.5 Hz, 1H, ArH), 7.93 (*d*, *^3^J* = 9.0 Hz, 1H, quinol), 7.82 (*ddd*, *^3^J* = 8.4, *^3^J* = 6.9 Hz, 1H, quinol), 7.73 (*dd*, *^3^J* = 7.9, 1H, ArH), 7.67 (*ddd*, *^3^J* = 8.1, *^3^J* = 6.9 Hz, 1H, quinol), 7.35 (*d*, *^3^J* = 7.9 Hz, 1H, quinol), 2.65 (*s*, 3H, CH_3_), 2.55 (*s*, 3H, COCH_3_). **^13^C NMR (100 MHz, CDCl_3_): δ** 197.8 (C_quat_), 162.2 (C_quat_), 149.5 (C_quat_), 146.2 (C_quat_), 138.0 (C_quat_), 136.2 (C_quat_), 133.5 (C_quat_), 130.7 (C_quat_), 130.4 (+), 129.8 (+), 129.5 (+), 128.3 (+), 127.8 (+), 124.1 (+), 121.5 (+), 118.6 (+), 26.7 (+), 18.0 (+).

***N*** **-(5-acetyl-2-bromo­phen­yl)quinoline-2-carboxamide (II):** Yellow solid (0.700 g, yield quant, *R*_f_ PE/EA 2:1 0.60). **^1^H NMR (400 MHz, CDCl_3_): δ** 9.32 (*s*, 1H), 8.40 (*d*, *^3^J* = 3.0 Hz, 2H), 8.23 (*d*, *^3^J* = 8.5 Hz, 1H), 7.94 (*d*, *^3^J* = 8.8 Hz, 1H), 7.83 (*t*, *^3^J* = 7.0 Hz, 1H), 7.69 (*m*, 3H), 2.68 (*s*, 3H, COCH_3_). **^13^C NMR (100 MHz, CDCl_3_): δ** 197.3 (C_quat_), 162.6 (C_quat_), 149.1 (C_quat_), 146.3 (C_quat_), 138.2 (C_quat_), 137.2 (C_quat_), 136.3 (C_quat_), 132.8 (+), 130.6 (+), 130.5 (+), 130.6 (+), 128.5 (+), 127.8 (+), 124.1 (+), 121.1 (+), 118.9 (+), 118.5 (+), 26.7 (+).

***N*** **-(5-acetyl-2-ethynylphen­yl)quinoline-2-carboxamide (III):** Light-brown solid (0.700 g, yield quant, *R*_f_ PE/EA 2:1 0.20). **^1^H NMR (400 MHz, CDCl_3_): δ** 9.36 (*d*, *^3^J* = 1.6 Hz, 1H), 8.40 (*s*, 2H), 8.15 (*d*, *^3^J* = 8.5 Hz, 1H), 7.94 (*d*, *^3^J* = 8.2 Hz, 1H), 7.82 (*dd*, *^3^J* = 11.2, 4.2 Hz, 1H), 7.73 (*dd*, *^3^J* = 8.1, *^3^J* = 1.7 Hz, 1H), 7.71 (*m*, 1H), 7.63 (*d*, *J* = 8.1 Hz, 1H), 3.87 (*s*, 1H, CCH), 2.70 (*s*, 3H, CH_3_). **^13^C NMR (100 MHz, CDCl_3_): δ** = 197.6 (C_quat_), 162.8 (C_quat_), 149.2 (C_quat_), 146.3 (C_quat_), 140.1 (C_quat_), 138.0 (C_quat_), 132.5 (+), 130.4 (+), 129.9 (+), 129.5 (+), 128.4 (+), 127.8 (+), 122.6 (+), 119.1 (+), 118.6 (+), 115.7 (+), 87.1 (C_quat_), 79.0 (+), 26.8 (+).

## Refinement   

Crystal data, data collection and structure refinement details are summarized in Table 4 ▸. All non-hydrogen atoms were refined anisotropically. Hydrogen-atom positions were calculated geometrically and refined using the riding model. N–H = 0.86 Å, C—H = 0.96 Å for methyl H atoms and 0.93 Å for all other; *U*_iso_(H) = 1.2*U*_eq_(C,N) or 1.5*U*_eq_(C_meth­yl_).

## Supplementary Material

Crystal structure: contains datablock(s) I, II, III. DOI: 10.1107/S2056989017006272/lh5839sup1.cif

Structure factors: contains datablock(s) I. DOI: 10.1107/S2056989017006272/lh5839Isup2.hkl

Structure factors: contains datablock(s) II. DOI: 10.1107/S2056989017006272/lh5839IIsup3.hkl

Structure factors: contains datablock(s) III. DOI: 10.1107/S2056989017006272/lh5839IIIsup4.hkl

Supporting information file. DOI: 10.1107/S2056989017006272/lh5839Isup5.cml

Supporting information file. DOI: 10.1107/S2056989017006272/lh5839IIsup6.cml

Supporting information file. DOI: 10.1107/S2056989017006272/lh5839IIIsup7.cml

CCDC references: 1546038, 1546037, 1546036

Additional supporting information:  crystallographic information; 3D view; checkCIF report

## Figures and Tables

**Figure 2:**
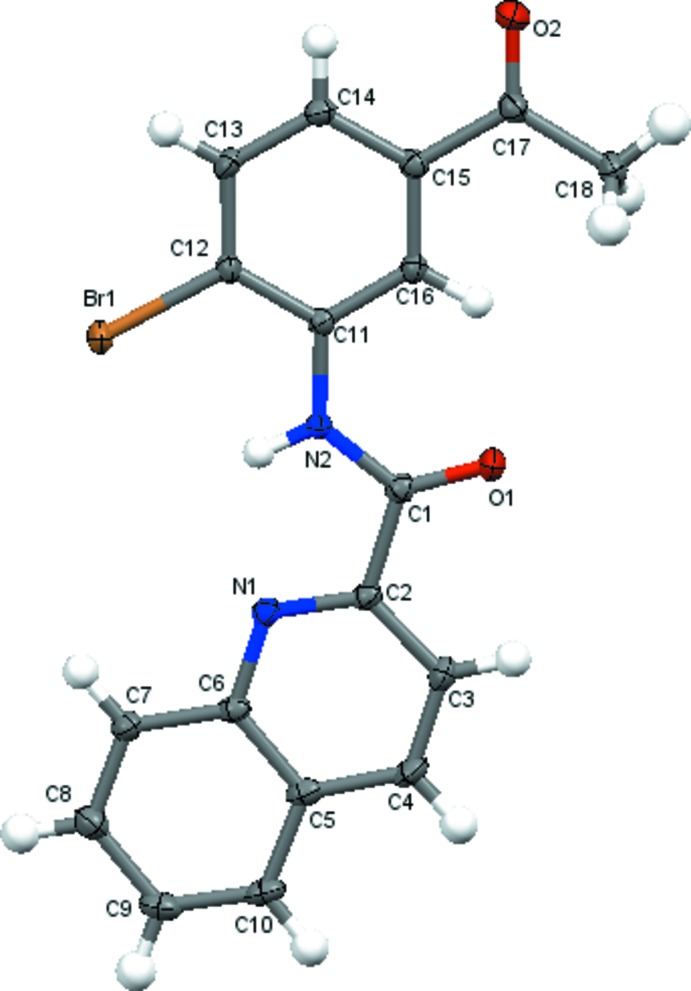
The mol­ecular structure of (II), with the atom-numbering scheme and displacement ellipsoids drawn at the 50% probability level.

**Figure 3:**
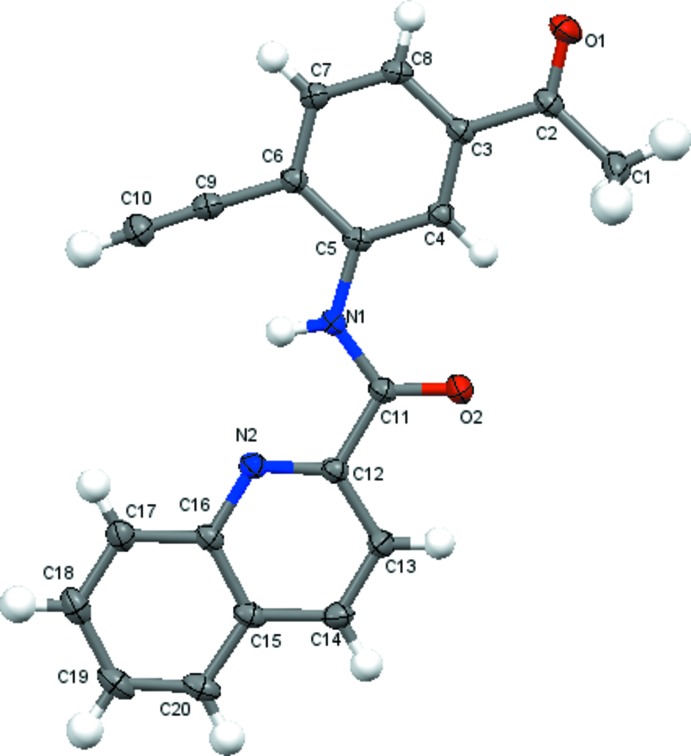
The mol­ecular structure of (III), with the-atom numbering scheme and displacement ellipsoids drawn at the 50% probability level.

**Table 1:** Hydrogen-bond geometry (Å, °) for (I)

*D*—H⋯*A*	*D*—H	H⋯*A*	*D*⋯*A*	*D*—H⋯*A*
N2—H2⋯N1	0.86	2.15	2.619 (2)	114
C17—H17⋯O1^i^	0.96	2.49	3.424 (2)	164

Symmetry code: (i) 
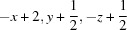
.

**Table 2:** Hydrogen-bond geometry (Å, °) for (II)

*D*—H⋯*A*	*D*—H	H⋯*A*	*D*⋯*A*	*D*—H⋯*A*
N2—H2⋯N1	0.86	2.19	2.629 (2)	112
C3—H3⋯O1^i^	0.93	2.55	3.410 (2)	154
C18—H18⋯O2^ii^	0.96	2.49	3.444 (2)	171
N2—H2⋯Br1	0.86	2.58	3.081 (1)	118

Symmetry codes: (i) 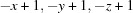; (ii) 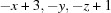.

**Table 3:** Hydrogen-bond geometry (Å, °) for (III)

*D*—H⋯*A*	*D*—H	H⋯*A*	*D*⋯*A*	*D*—H⋯*A*
N1—H1⋯N2	0.86	2.23	2.666 (2)	111
C10—H10⋯O2^i^	0.93	2.36	3.103 (2)	136

Symmetry code: (i) 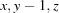.

**Table 4:** Experimental details

	(I)	(II)	(III)
Crystal data			
Chemical formula	C_19_H_16_N_2_O_2_	C_18_H_13_BrN_2_O_2_	C_20_H_14_N_2_O_2_
*M*_r_	304.34	369.21	314.33
Crystal system, space group	Monoclinic, *P*2_1_/*c*	Triclinic, *P* 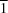	Triclinic, *P* 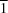
Temperature (K)	123	123	123
*a*, *b*, *c* (Å)	4.5787 (2), 14.7986 (7), 22.3732 (12)	4.29848 (12), 11.6353 (3), 15.5888 (4)	7.3075 (6), 8.2605 (4), 13.8196 (9)
α, β, γ (°)	90, 92.130 (5), 90	103.788 (2), 95.515 (2), 96.195 (2)	92.734 (5), 100.608 (6), 108.989 (6)
*V* (Å^3^)	1514.93 (12)	746.76 (3)	770.11 (10)
*Z*	4	2	2
Radiation type	Cu *K*α	Cu *K*α	Mo *K*α
μ (mm^−1^)	0.71	3.85	0.09
Crystal size (mm)	0.33 × 0.12 × 0.07	0.65 × 0.10 × 0.06	0.19 × 0.08 × 0.05

Data collection			
Diffractometer	Agilent TitanS2 GV1000	Agilent TitanS2 GV1000	Agilent SuperNova Single source at offset, Eos
Absorption correction	Analytical [*CrysAlis PRO* (Rigaku OD, 2015 ▸), based on expressions derived by Clark & Reid (1995 ▸)]	Gaussian [*CrysAlis PRO* (Rigaku OD, 2015 ▸), based on expressions derived by Clark & Reid (1995 ▸)]	Analytical [*CrysAlis PRO* (Rigaku OD, 2015 ▸), based on expressions derived by Clark & Reid (1995 ▸)]
*T*_min_, *T*_max_	0.869, 0.958	0.540, 0.900	0.987, 0.996
No. of measured, independent and observed [*I* > 2σ(*I*)] reflections	6056, 2935, 2562	12899, 2966, 2870	20693, 5173, 3687
*R*_int_	0.020	0.033	0.060
(sin θ/λ)_max_ (Å^−1^)	0.625	0.622	0.753

Refinement			
*R*[*F* ^2^ > 2σ(*F* ^2^)], *wR*(*F* ^2^), *S*	0.038, 0.106, 1.04	0.024, 0.065, 1.05	0.056, 0.151, 1.04
No. of reflections	2935	2966	5173
No. of parameters	210	210	218
H-atom treatment	H-atom parameters constrained	H-atom parameters constrained	H-atom parameters constrained
Δρ_max_, Δρ_min_ (e Å^−3^)	0.20, −0.21	0.48, −0.65	0.42, −0.26

Computer programs: *CrysAlis PRO* (Rigaku OD, 2015 ▸), *SHELXT* (Sheldrick, 2015*a* ▸), *SHELXL2014* (Sheldrick, 2015*b* ▸) and *OLEX2* (Dolomanov *et al.*, 2009 ▸).

## supplementary crystallographic information

### (I) N-(5-Acetyl-2-methylphenyl)quinoline-2-carboxamide Crystal data

**Table:**

C_19_H_16_N_2_O_2_	*F*(000) = 640
*M**_r_* = 304.34	*D*_x_ = 1.334 Mg m^−^^3^
Monoclinic, *P*2_1_/*c*	Cu *K*α radiation, λ = 1.54184 Å
*a* = 4.5787 (2) Å	Cell parameters from 3477 reflections
*b* = 14.7986 (7) Å	θ = 6.0–74.2°
*c* = 22.3732 (12) Å	µ = 0.71 mm^−^^1^
β = 92.130 (5)°	*T* = 123 K
*V* = 1514.93 (12) Å^3^	Block, dark gray
*Z* = 4	0.33 × 0.12 × 0.07 mm

### (I) N-(5-Acetyl-2-methylphenyl)quinoline-2-carboxamide Data collection

**Table:**

Agilent TitanS2 GV1000 diffractometer	2935 independent reflections
Radiation source: gradient vaccum rotating-anode X-ray tube, GV1000 (Cu) X-ray Source	2562 reflections with *I* > 2σ(*I*)
Mirror monochromator	*R*_int_ = 0.020
Detector resolution: 4.1818 pixels mm^-1^	θ_max_ = 74.4°, θ_min_ = 3.6°
ω scans	*h* = −5→5
Absorption correction: analytical [CrysAlis PRO (Rigaku OD, 2015), based on expressions derived by Clark & Reid (1995)]	*k* = −18→17
*T*_min_ = 0.869, *T*_max_ = 0.958	*l* = −27→21
6056 measured reflections	

### (I) N-(5-Acetyl-2-methylphenyl)quinoline-2-carboxamide Refinement

**Table:**

Refinement on *F*^2^	0 restraints
Least-squares matrix: full	Hydrogen site location: inferred from neighbouring sites
*R*[*F*^2^ > 2σ(*F*^2^)] = 0.038	H-atom parameters constrained
*wR*(*F*^2^) = 0.106	*w* = 1/[σ^2^(*F*_o_^2^) + (0.0608*P*)^2^ + 0.2546*P*] where *P* = (*F*_o_^2^ + 2*F*_c_^2^)/3
*S* = 1.04	(Δ/σ)_max_ < 0.001
2935 reflections	Δρ_max_ = 0.20 e Å^−^^3^
210 parameters	Δρ_min_ = −0.21 e Å^−^^3^

### (I) N-(5-Acetyl-2-methylphenyl)quinoline-2-carboxamide Special details

**Table:**

Geometry. All esds (except the esd in the dihedral angle between two l.s. planes) are estimated using the full covariance matrix. The cell esds are taken into account individually in the estimation of esds in distances, angles and torsion angles; correlations between esds in cell parameters are only used when they are defined by crystal symmetry. An approximate (isotropic) treatment of cell esds is used for estimating esds involving l.s. planes.

### (I) N-(5-Acetyl-2-methylphenyl)quinoline-2-carboxamide Fractional atomic coordinates and isotropic or equivalent isotropic displacement parameters (Å^2^)

**Table:**

	*x*	*y*	*z*	*U*_iso_*/*U*_eq_	
O1	0.59998 (18)	0.50983 (6)	0.24602 (4)	0.0250 (2)	
O2	1.4993 (2)	0.62002 (8)	0.05885 (5)	0.0410 (3)	
N2	0.7067 (2)	0.65842 (7)	0.26763 (5)	0.0217 (2)	
H2	0.6628	0.7001	0.2925	0.026*	
N1	0.3450 (2)	0.65936 (7)	0.35561 (5)	0.0223 (2)	
C10	0.5705 (2)	0.57873 (8)	0.27553 (5)	0.0197 (2)	
C9	0.3690 (2)	0.58150 (8)	0.32767 (5)	0.0200 (2)	
C16	1.0023 (2)	0.62534 (8)	0.18077 (5)	0.0225 (3)	
H16	0.9290	0.5668	0.1784	0.027*	
C11	0.9095 (2)	0.68391 (8)	0.22502 (5)	0.0205 (3)	
C12	1.0191 (2)	0.77271 (8)	0.22953 (6)	0.0223 (3)	
C8	0.2199 (2)	0.50181 (8)	0.34310 (6)	0.0229 (3)	
H8	0.2444	0.4485	0.3219	0.028*	
C15	1.2058 (2)	0.65473 (8)	0.13999 (6)	0.0239 (3)	
C6	0.1639 (2)	0.66364 (9)	0.40278 (5)	0.0242 (3)	
C17	0.9276 (3)	0.83545 (8)	0.27837 (6)	0.0257 (3)	
H17A	0.7217	0.8470	0.2739	0.039*	
H17B	1.0329	0.8913	0.2758	0.039*	
H17C	0.9700	0.8080	0.3166	0.039*	
C13	1.2190 (3)	0.80052 (8)	0.18796 (6)	0.0252 (3)	
H13	1.2917	0.8592	0.1899	0.030*	
C14	1.3123 (3)	0.74284 (9)	0.14374 (6)	0.0268 (3)	
H14	1.4464	0.7630	0.1165	0.032*	
C7	0.0383 (3)	0.50538 (9)	0.39030 (6)	0.0260 (3)	
H7	−0.0619	0.4539	0.4018	0.031*	
C5	0.0036 (2)	0.58715 (9)	0.42139 (6)	0.0256 (3)	
C18	1.3186 (3)	0.59328 (9)	0.09298 (6)	0.0284 (3)	
C1	0.1359 (3)	0.74684 (10)	0.43341 (6)	0.0309 (3)	
H1	0.2429	0.7969	0.4218	0.037*	
C4	−0.1839 (3)	0.59647 (11)	0.47008 (6)	0.0337 (3)	
H4	−0.2897	0.5469	0.4830	0.040*	
C3	−0.2098 (3)	0.67783 (12)	0.49810 (6)	0.0395 (4)	
H3	−0.3356	0.6835	0.5296	0.047*	
C2	−0.0478 (3)	0.75369 (11)	0.47989 (7)	0.0371 (3)	
H2A	−0.0665	0.8085	0.4997	0.045*	
C19	1.2077 (4)	0.49817 (10)	0.08911 (8)	0.0446 (4)	
H19A	1.2992	0.4673	0.0570	0.067*	
H19B	0.9998	0.4988	0.0818	0.067*	
H19C	1.2530	0.4674	0.1261	0.067*	

### (I) N-(5-Acetyl-2-methylphenyl)quinoline-2-carboxamide Atomic displacement parameters (Å^2^)

**Table:**

	*U*^11^	*U*^22^	*U*^33^	*U*^12^	*U*^13^	*U*^23^
O1	0.0249 (4)	0.0214 (4)	0.0291 (5)	0.0003 (3)	0.0072 (3)	−0.0016 (3)
O2	0.0414 (5)	0.0441 (6)	0.0388 (6)	−0.0028 (4)	0.0208 (4)	0.0001 (5)
N2	0.0217 (5)	0.0199 (5)	0.0239 (5)	0.0002 (4)	0.0055 (4)	−0.0005 (4)
N1	0.0195 (5)	0.0248 (5)	0.0226 (5)	0.0028 (4)	0.0008 (4)	0.0000 (4)
C10	0.0152 (5)	0.0210 (5)	0.0228 (6)	0.0018 (4)	0.0002 (4)	0.0018 (4)
C9	0.0163 (5)	0.0233 (5)	0.0202 (6)	0.0018 (4)	−0.0006 (4)	0.0015 (4)
C16	0.0197 (5)	0.0229 (5)	0.0250 (6)	0.0013 (4)	0.0017 (4)	0.0028 (5)
C11	0.0159 (5)	0.0229 (5)	0.0226 (6)	0.0009 (4)	0.0008 (4)	0.0050 (4)
C12	0.0188 (5)	0.0222 (6)	0.0258 (6)	0.0021 (4)	−0.0014 (4)	0.0035 (5)
C8	0.0201 (5)	0.0246 (6)	0.0240 (6)	−0.0003 (4)	0.0007 (4)	0.0011 (5)
C15	0.0200 (5)	0.0277 (6)	0.0242 (6)	0.0030 (4)	0.0028 (4)	0.0047 (5)
C6	0.0197 (5)	0.0316 (6)	0.0210 (6)	0.0051 (5)	−0.0010 (4)	0.0006 (5)
C17	0.0275 (6)	0.0211 (5)	0.0286 (6)	−0.0011 (5)	0.0017 (5)	0.0013 (5)
C13	0.0217 (5)	0.0228 (5)	0.0312 (7)	−0.0023 (4)	0.0002 (5)	0.0072 (5)
C14	0.0216 (6)	0.0308 (6)	0.0283 (7)	0.0000 (5)	0.0050 (5)	0.0081 (5)
C7	0.0201 (5)	0.0322 (6)	0.0256 (6)	−0.0028 (5)	0.0008 (5)	0.0050 (5)
C5	0.0185 (5)	0.0377 (7)	0.0205 (6)	0.0034 (5)	−0.0002 (4)	0.0038 (5)
C18	0.0246 (6)	0.0340 (7)	0.0268 (6)	0.0034 (5)	0.0057 (5)	0.0039 (5)
C1	0.0310 (6)	0.0346 (7)	0.0270 (7)	0.0079 (5)	−0.0014 (5)	−0.0046 (5)
C4	0.0243 (6)	0.0522 (8)	0.0249 (7)	0.0045 (6)	0.0047 (5)	0.0061 (6)
C3	0.0304 (7)	0.0648 (10)	0.0236 (7)	0.0138 (7)	0.0054 (5)	−0.0011 (7)
C2	0.0360 (7)	0.0479 (8)	0.0273 (7)	0.0147 (6)	−0.0015 (5)	−0.0097 (6)
C19	0.0536 (9)	0.0348 (8)	0.0472 (9)	−0.0030 (6)	0.0261 (7)	−0.0092 (7)

### (I) N-(5-Acetyl-2-methylphenyl)quinoline-2-carboxamide Geometric parameters (Å, º)

**Table:**

O1—C10	1.2249 (15)	C17—H17A	0.9600
O2—C18	1.2129 (17)	C17—H17B	0.9600
N2—H2	0.8600	C17—H17C	0.9600
N2—C10	1.3490 (15)	C13—H13	0.9300
N2—C11	1.4068 (15)	C13—C14	1.3861 (19)
N1—C9	1.3175 (15)	C14—H14	0.9300
N1—C6	1.3674 (16)	C7—H7	0.9300
C10—C9	1.5142 (16)	C7—C5	1.4075 (19)
C9—C8	1.4120 (16)	C5—C4	1.4188 (18)
C16—H16	0.9300	C18—C19	1.498 (2)
C16—C11	1.3940 (17)	C1—H1	0.9300
C16—C15	1.3973 (17)	C1—C2	1.365 (2)
C11—C12	1.4090 (16)	C4—H4	0.9300
C12—C17	1.5051 (17)	C4—C3	1.365 (2)
C12—C13	1.3912 (17)	C3—H3	0.9300
C8—H8	0.9300	C3—C2	1.414 (2)
C8—C7	1.3694 (17)	C2—H2A	0.9300
C15—C14	1.3937 (18)	C19—H19A	0.9600
C15—C18	1.4970 (18)	C19—H19B	0.9600
C6—C5	1.4200 (18)	C19—H19C	0.9600
C6—C1	1.4172 (18)		
			
C10—N2—H2	115.0	C12—C13—H13	119.2
C10—N2—C11	130.05 (10)	C14—C13—C12	121.53 (11)
C11—N2—H2	115.0	C14—C13—H13	119.2
C9—N1—C6	118.07 (10)	C15—C14—H14	119.9
O1—C10—N2	126.59 (11)	C13—C14—C15	120.17 (11)
O1—C10—C9	121.36 (10)	C13—C14—H14	119.9
N2—C10—C9	112.04 (10)	C8—C7—H7	120.1
N1—C9—C10	116.96 (10)	C8—C7—C5	119.81 (11)
N1—C9—C8	124.38 (11)	C5—C7—H7	120.1
C8—C9—C10	118.66 (10)	C7—C5—C6	118.13 (11)
C11—C16—H16	120.1	C7—C5—C4	123.06 (12)
C11—C16—C15	119.89 (11)	C4—C5—C6	118.81 (12)
C15—C16—H16	120.1	O2—C18—C15	120.37 (13)
N2—C11—C12	116.35 (10)	O2—C18—C19	120.53 (13)
C16—C11—N2	122.74 (11)	C15—C18—C19	119.10 (11)
C16—C11—C12	120.91 (11)	C6—C1—H1	119.9
C11—C12—C17	121.28 (11)	C2—C1—C6	120.15 (14)
C13—C12—C11	118.00 (11)	C2—C1—H1	119.9
C13—C12—C17	120.71 (11)	C5—C4—H4	119.9
C9—C8—H8	121.0	C3—C4—C5	120.25 (14)
C7—C8—C9	118.00 (11)	C3—C4—H4	119.9
C7—C8—H8	121.0	C4—C3—H3	119.6
C16—C15—C18	121.71 (11)	C4—C3—C2	120.85 (13)
C14—C15—C16	119.50 (12)	C2—C3—H3	119.6
C14—C15—C18	118.77 (11)	C1—C2—C3	120.34 (14)
N1—C6—C5	121.61 (11)	C1—C2—H2A	119.8
N1—C6—C1	118.80 (12)	C3—C2—H2A	119.8
C1—C6—C5	119.59 (12)	C18—C19—H19A	109.5
C12—C17—H17A	109.5	C18—C19—H19B	109.5
C12—C17—H17B	109.5	C18—C19—H19C	109.5
C12—C17—H17C	109.5	H19A—C19—H19B	109.5
H17A—C17—H17B	109.5	H19A—C19—H19C	109.5
H17A—C17—H17C	109.5	H19B—C19—H19C	109.5
H17B—C17—H17C	109.5		
			
O1—C10—C9—N1	−178.04 (10)	C11—C16—C15—C14	−0.49 (18)
O1—C10—C9—C8	1.81 (16)	C11—C16—C15—C18	178.04 (11)
N2—C10—C9—N1	2.76 (14)	C11—C12—C13—C14	−0.89 (17)
N2—C10—C9—C8	−177.40 (10)	C12—C13—C14—C15	0.12 (19)
N2—C11—C12—C17	1.36 (16)	C8—C7—C5—C6	−0.83 (17)
N2—C11—C12—C13	−179.66 (10)	C8—C7—C5—C4	178.99 (11)
N1—C9—C8—C7	−0.09 (18)	C15—C16—C11—N2	−179.62 (10)
N1—C6—C5—C7	0.80 (17)	C15—C16—C11—C12	−0.31 (17)
N1—C6—C5—C4	−179.03 (11)	C6—N1—C9—C10	179.88 (9)
N1—C6—C1—C2	178.65 (12)	C6—N1—C9—C8	0.04 (17)
C10—N2—C11—C16	0.95 (19)	C6—C5—C4—C3	0.34 (18)
C10—N2—C11—C12	−178.39 (11)	C6—C1—C2—C3	0.4 (2)
C10—C9—C8—C7	−179.93 (10)	C17—C12—C13—C14	178.09 (11)
C9—N1—C6—C5	−0.41 (16)	C14—C15—C18—O2	−0.05 (19)
C9—N1—C6—C1	179.89 (11)	C14—C15—C18—C19	179.31 (13)
C9—C8—C7—C5	0.49 (17)	C7—C5—C4—C3	−179.48 (12)
C16—C11—C12—C17	−177.99 (11)	C5—C6—C1—C2	−1.07 (19)
C16—C11—C12—C13	0.99 (17)	C5—C4—C3—C2	−1.0 (2)
C16—C15—C14—C13	0.59 (18)	C18—C15—C14—C13	−177.99 (11)
C16—C15—C18—O2	−178.60 (12)	C1—C6—C5—C7	−179.50 (11)
C16—C15—C18—C19	0.76 (19)	C1—C6—C5—C4	0.67 (17)
C11—N2—C10—O1	0.6 (2)	C4—C3—C2—C1	0.6 (2)
C11—N2—C10—C9	179.74 (10)		

### (I) N-(5-Acetyl-2-methylphenyl)quinoline-2-carboxamide Hydrogen-bond geometry (Å, º)

**Table:**

*D*—H···*A*	*D*—H	H···*A*	*D*···*A*	*D*—H···*A*
N2—H2···N1	0.86	2.15	2.619 (2)	114
C17—H17···O1^i^	0.96	2.49	3.424 (2)	164

Symmetry code: (i) −*x*+2, *y*+1/2, −*z*+1/2.

### (II) N-(5-Acetyl-2-bromophenyl)quinoline-2-carboxamide Crystal data

**Table:**

C_18_H_13_BrN_2_O_2_	*Z* = 2
*M**_r_* = 369.21	*F*(000) = 372
Triclinic, *P*1	*D*_x_ = 1.642 Mg m^−^^3^
*a* = 4.29848 (12) Å	Cu *K*α radiation, λ = 1.54184 Å
*b* = 11.6353 (3) Å	Cell parameters from 10806 reflections
*c* = 15.5888 (4) Å	θ = 3.9–73.8°
α = 103.788 (2)°	µ = 3.85 mm^−^^1^
β = 95.515 (2)°	*T* = 123 K
γ = 96.195 (2)°	Plank, clear colourless
*V* = 746.76 (3) Å^3^	0.65 × 0.10 × 0.06 mm

### (II) N-(5-Acetyl-2-bromophenyl)quinoline-2-carboxamide Data collection

**Table:**

Agilent TitanS2 GV1000 diffractometer	2966 independent reflections
Radiation source: gradient vaccum rotating-anode X-ray tube, GV1000 (Cu) X-ray Source	2870 reflections with *I* > 2σ(*I*)
Mirror monochromator	*R*_int_ = 0.033
Detector resolution: 4.1818 pixels mm^-1^	θ_max_ = 73.7°, θ_min_ = 2.9°
ω scans	*h* = −5→5
Absorption correction: gaussian [CrysAlis PRO (Rigaku OD, 2015), based on expressions derived by Clark & Reid (1995)]	*k* = −13→14
*T*_min_ = 0.540, *T*_max_ = 0.900	*l* = −19→19
12899 measured reflections	

### (II) N-(5-Acetyl-2-bromophenyl)quinoline-2-carboxamide Refinement

**Table:**

Refinement on *F*^2^	Hydrogen site location: inferred from neighbouring sites
Least-squares matrix: full	H-atom parameters constrained
*R*[*F*^2^ > 2σ(*F*^2^)] = 0.024	*w* = 1/[σ^2^(*F*_o_^2^) + (0.039*P*)^2^ + 0.3702*P*] where *P* = (*F*_o_^2^ + 2*F*_c_^2^)/3
*wR*(*F*^2^) = 0.065	(Δ/σ)_max_ = 0.001
*S* = 1.05	Δρ_max_ = 0.48 e Å^−^^3^
2966 reflections	Δρ_min_ = −0.65 e Å^−^^3^
210 parameters	Extinction correction: SHELXL2014 (Sheldrick, 2015b), Fc^*^=kFc[1+0.001xFc^2^λ^3^/sin(2θ)]^-1/4^
0 restraints	Extinction coefficient: 0.0018 (3)

### (II) N-(5-Acetyl-2-bromophenyl)quinoline-2-carboxamide Special details

**Table:**

Geometry. All esds (except the esd in the dihedral angle between two l.s. planes) are estimated using the full covariance matrix. The cell esds are taken into account individually in the estimation of esds in distances, angles and torsion angles; correlations between esds in cell parameters are only used when they are defined by crystal symmetry. An approximate (isotropic) treatment of cell esds is used for estimating esds involving l.s. planes.

### (II) N-(5-Acetyl-2-bromophenyl)quinoline-2-carboxamide Fractional atomic coordinates and isotropic or equivalent isotropic displacement parameters (Å^2^)

**Table:**

	*x*	*y*	*z*	*U*_iso_*/*U*_eq_	
Br1	0.61589 (4)	0.18439 (2)	0.04683 (2)	0.02135 (9)	
O1	0.5793 (3)	0.36128 (11)	0.39598 (8)	0.0221 (3)	
O2	1.3517 (3)	−0.04850 (12)	0.36241 (9)	0.0289 (3)	
N2	0.5689 (3)	0.29917 (12)	0.24400 (9)	0.0152 (3)	
H2	0.4949	0.3149	0.1955	0.018*	
N1	0.2021 (3)	0.45877 (12)	0.22055 (9)	0.0155 (3)	
C11	0.7457 (4)	0.20417 (15)	0.23433 (11)	0.0144 (3)	
C1	0.4988 (4)	0.37012 (15)	0.32082 (11)	0.0161 (3)	
C6	0.0260 (4)	0.54368 (15)	0.20390 (11)	0.0159 (3)	
C15	1.0588 (4)	0.07376 (15)	0.29489 (11)	0.0161 (3)	
C12	0.7872 (4)	0.13977 (15)	0.14896 (11)	0.0161 (3)	
C2	0.3045 (4)	0.46475 (15)	0.30427 (11)	0.0156 (3)	
C16	0.8882 (4)	0.16983 (15)	0.30694 (11)	0.0158 (3)	
H16	0.8683	0.2121	0.3644	0.019*	
C13	0.9537 (4)	0.04208 (16)	0.13641 (11)	0.0185 (3)	
H13	0.9742	−0.0006	0.0791	0.022*	
C3	0.2451 (4)	0.55339 (16)	0.37799 (11)	0.0202 (4)	
H3	0.3202	0.5528	0.4358	0.024*	
C14	1.0880 (4)	0.00874 (16)	0.20912 (11)	0.0183 (3)	
H14	1.1978	−0.0569	0.2010	0.022*	
C18	1.2121 (4)	0.11879 (17)	0.46469 (11)	0.0219 (4)	
H18A	0.9983	0.1174	0.4778	0.033*	
H18B	1.2985	0.1991	0.4666	0.033*	
H18C	1.3353	0.0903	0.5080	0.033*	
C17	1.2189 (4)	0.03975 (16)	0.37334 (11)	0.0187 (3)	
C7	−0.0919 (4)	0.53725 (16)	0.11481 (11)	0.0190 (3)	
H7	−0.0509	0.4752	0.0692	0.023*	
C8	−0.2654 (4)	0.62145 (17)	0.09514 (12)	0.0231 (4)	
H8	−0.3398	0.6169	0.0362	0.028*	
C9	−0.3322 (4)	0.71549 (17)	0.16407 (13)	0.0241 (4)	
H9	−0.4509	0.7723	0.1501	0.029*	
C4	0.0744 (4)	0.63973 (16)	0.36164 (12)	0.0221 (4)	
H4	0.0344	0.7000	0.4087	0.026*	
C10	−0.2242 (4)	0.72361 (16)	0.25081 (13)	0.0220 (4)	
H10	−0.2703	0.7858	0.2955	0.026*	
C5	−0.0422 (4)	0.63791 (15)	0.27334 (12)	0.0179 (3)	

### (II) N-(5-Acetyl-2-bromophenyl)quinoline-2-carboxamide Atomic displacement parameters (Å^2^)

**Table:**

	*U*^11^	*U*^22^	*U*^33^	*U*^12^	*U*^13^	*U*^23^
Br1	0.03126 (13)	0.02289 (13)	0.01153 (11)	0.00980 (8)	0.00226 (7)	0.00496 (8)
O1	0.0320 (7)	0.0228 (7)	0.0123 (6)	0.0102 (5)	0.0028 (5)	0.0033 (5)
O2	0.0414 (8)	0.0268 (7)	0.0214 (6)	0.0190 (6)	0.0013 (6)	0.0062 (5)
N2	0.0197 (7)	0.0159 (7)	0.0116 (6)	0.0069 (5)	0.0031 (5)	0.0042 (5)
N1	0.0181 (6)	0.0141 (7)	0.0149 (6)	0.0026 (5)	0.0049 (5)	0.0034 (5)
C11	0.0159 (7)	0.0133 (8)	0.0145 (7)	0.0019 (6)	0.0036 (6)	0.0039 (6)
C1	0.0172 (7)	0.0155 (8)	0.0155 (8)	0.0016 (6)	0.0039 (6)	0.0032 (6)
C6	0.0162 (7)	0.0138 (8)	0.0189 (8)	0.0031 (6)	0.0063 (6)	0.0046 (6)
C15	0.0171 (7)	0.0164 (8)	0.0155 (8)	0.0019 (6)	0.0027 (6)	0.0053 (6)
C12	0.0194 (8)	0.0181 (8)	0.0113 (7)	0.0022 (6)	0.0018 (6)	0.0049 (6)
C2	0.0179 (7)	0.0138 (8)	0.0156 (8)	0.0026 (6)	0.0056 (6)	0.0029 (6)
C16	0.0185 (7)	0.0166 (8)	0.0126 (7)	0.0028 (6)	0.0038 (6)	0.0036 (6)
C13	0.0238 (8)	0.0173 (9)	0.0138 (8)	0.0047 (7)	0.0050 (6)	0.0007 (6)
C3	0.0250 (8)	0.0211 (9)	0.0138 (8)	0.0044 (7)	0.0041 (6)	0.0017 (7)
C14	0.0212 (8)	0.0148 (8)	0.0196 (8)	0.0054 (6)	0.0044 (7)	0.0033 (7)
C18	0.0300 (9)	0.0223 (9)	0.0156 (8)	0.0090 (7)	0.0026 (7)	0.0070 (7)
C17	0.0211 (8)	0.0196 (9)	0.0177 (8)	0.0052 (7)	0.0040 (6)	0.0074 (7)
C7	0.0234 (8)	0.0170 (9)	0.0176 (8)	0.0059 (7)	0.0055 (7)	0.0038 (7)
C8	0.0261 (9)	0.0232 (10)	0.0229 (9)	0.0069 (7)	0.0028 (7)	0.0098 (7)
C9	0.0238 (9)	0.0181 (9)	0.0336 (10)	0.0086 (7)	0.0051 (7)	0.0096 (8)
C4	0.0270 (9)	0.0180 (9)	0.0191 (8)	0.0062 (7)	0.0068 (7)	−0.0023 (7)
C10	0.0235 (8)	0.0135 (8)	0.0291 (9)	0.0060 (7)	0.0085 (7)	0.0015 (7)
C5	0.0183 (8)	0.0139 (8)	0.0215 (8)	0.0027 (6)	0.0071 (6)	0.0026 (7)

### (II) N-(5-Acetyl-2-bromophenyl)quinoline-2-carboxamide Geometric parameters (Å, º)

**Table:**

Br1—C12	1.8968 (16)	C13—H13	0.9300
O1—C1	1.221 (2)	C13—C14	1.379 (2)
O2—C17	1.213 (2)	C3—H3	0.9300
N2—H2	0.8600	C3—C4	1.363 (3)
N2—C11	1.395 (2)	C14—H14	0.9300
N2—C1	1.363 (2)	C18—H18A	0.9600
N1—C6	1.365 (2)	C18—H18B	0.9600
N1—C2	1.320 (2)	C18—H18C	0.9600
C11—C12	1.401 (2)	C18—C17	1.505 (2)
C11—C16	1.397 (2)	C7—H7	0.9300
C1—C2	1.506 (2)	C7—C8	1.366 (3)
C6—C7	1.413 (2)	C8—H8	0.9300
C6—C5	1.423 (2)	C8—C9	1.415 (3)
C15—C16	1.388 (2)	C9—H9	0.9300
C15—C14	1.395 (2)	C9—C10	1.365 (3)
C15—C17	1.501 (2)	C4—H4	0.9300
C12—C13	1.392 (2)	C4—C5	1.413 (3)
C2—C3	1.414 (2)	C10—H10	0.9300
C16—H16	0.9300	C10—C5	1.419 (3)
			
C11—N2—H2	116.0	C15—C14—H14	120.1
C1—N2—H2	116.0	C13—C14—C15	119.79 (16)
C1—N2—C11	128.05 (14)	C13—C14—H14	120.1
C2—N1—C6	117.86 (14)	H18A—C18—H18B	109.5
N2—C11—C12	119.73 (14)	H18A—C18—H18C	109.5
N2—C11—C16	122.69 (14)	H18B—C18—H18C	109.5
C16—C11—C12	117.58 (15)	C17—C18—H18A	109.5
O1—C1—N2	125.78 (16)	C17—C18—H18B	109.5
O1—C1—C2	121.70 (15)	C17—C18—H18C	109.5
N2—C1—C2	112.53 (14)	O2—C17—C15	120.30 (16)
N1—C6—C7	118.71 (15)	O2—C17—C18	121.59 (16)
N1—C6—C5	122.01 (15)	C15—C17—C18	118.10 (15)
C7—C6—C5	119.28 (16)	C6—C7—H7	119.7
C16—C15—C14	120.08 (15)	C8—C7—C6	120.57 (16)
C16—C15—C17	120.76 (15)	C8—C7—H7	119.7
C14—C15—C17	119.14 (15)	C7—C8—H8	119.9
C11—C12—Br1	120.33 (13)	C7—C8—C9	120.21 (17)
C13—C12—Br1	118.19 (12)	C9—C8—H8	119.9
C13—C12—C11	121.48 (15)	C8—C9—H9	119.7
N1—C2—C1	116.81 (14)	C10—C9—C8	120.64 (17)
N1—C2—C3	124.47 (16)	C10—C9—H9	119.7
C3—C2—C1	118.73 (15)	C3—C4—H4	119.9
C11—C16—H16	119.4	C3—C4—C5	120.19 (16)
C15—C16—C11	121.17 (15)	C5—C4—H4	119.9
C15—C16—H16	119.4	C9—C10—H10	119.8
C12—C13—H13	120.1	C9—C10—C5	120.48 (16)
C14—C13—C12	119.84 (15)	C5—C10—H10	119.8
C14—C13—H13	120.1	C4—C5—C6	117.53 (16)
C2—C3—H3	121.0	C4—C5—C10	123.66 (16)
C4—C3—C2	117.93 (16)	C10—C5—C6	118.80 (16)
C4—C3—H3	121.0		
			
Br1—C12—C13—C14	178.46 (13)	C2—N1—C6—C7	−178.60 (15)
O1—C1—C2—N1	−173.05 (15)	C2—N1—C6—C5	1.4 (2)
O1—C1—C2—C3	7.2 (2)	C2—C3—C4—C5	1.1 (3)
N2—C11—C12—Br1	2.1 (2)	C16—C11—C12—Br1	−177.52 (12)
N2—C11—C12—C13	−177.97 (15)	C16—C11—C12—C13	2.5 (2)
N2—C11—C16—C15	179.08 (15)	C16—C15—C14—C13	1.7 (2)
N2—C1—C2—N1	7.0 (2)	C16—C15—C17—O2	175.63 (17)
N2—C1—C2—C3	−172.76 (15)	C16—C15—C17—C18	−5.1 (2)
N1—C6—C7—C8	−179.06 (16)	C3—C4—C5—C6	0.0 (3)
N1—C6—C5—C4	−1.3 (2)	C3—C4—C5—C10	179.32 (17)
N1—C6—C5—C10	179.38 (15)	C14—C15—C16—C11	−0.7 (2)
N1—C2—C3—C4	−1.0 (3)	C14—C15—C17—O2	−5.5 (2)
C11—N2—C1—O1	−0.7 (3)	C14—C15—C17—C18	173.78 (16)
C11—N2—C1—C2	179.29 (14)	C17—C15—C16—C11	178.22 (14)
C11—C12—C13—C14	−1.5 (3)	C17—C15—C14—C13	−177.24 (15)
C1—N2—C11—C12	178.73 (15)	C7—C6—C5—C4	178.70 (15)
C1—N2—C11—C16	−1.7 (3)	C7—C6—C5—C10	−0.7 (2)
C1—C2—C3—C4	178.68 (15)	C7—C8—C9—C10	0.1 (3)
C6—N1—C2—C1	−179.91 (14)	C8—C9—C10—C5	0.2 (3)
C6—N1—C2—C3	−0.2 (2)	C9—C10—C5—C6	0.1 (3)
C6—C7—C8—C9	−0.7 (3)	C9—C10—C5—C4	−179.22 (17)
C12—C11—C16—C15	−1.4 (2)	C5—C6—C7—C8	1.0 (3)
C12—C13—C14—C15	−0.6 (3)		

### (II) N-(5-Acetyl-2-bromophenyl)quinoline-2-carboxamide Hydrogen-bond geometry (Å, º)

**Table:**

*D*—H···*A*	*D*—H	H···*A*	*D*···*A*	*D*—H···*A*
N2—H2···N1	0.86	2.19	2.629 (2)	112
C3—H3···O1^i^	0.93	2.55	3.410 (2)	154
C18—H18···O2^ii^	0.96	2.49	3.444 (2)	171
N2—H2···Br1	0.86	2.58	3.081 (1)	118

Symmetry codes: (i) −*x*+1, −*y*+1, −*z*+1; (ii) −*x*+3, −*y*, −*z*+1.

### (III) N-(5-Acetyl-2-ethynylphenyl)quinoline-2-carboxamide Crystal data

**Table:**

C_20_H_14_N_2_O_2_	*Z* = 2
*M**_r_* = 314.33	*F*(000) = 328
Triclinic, *P*1	*D*_x_ = 1.356 Mg m^−^^3^
*a* = 7.3075 (6) Å	Mo *K*α radiation, λ = 0.71073 Å
*b* = 8.2605 (4) Å	Cell parameters from 5519 reflections
*c* = 13.8196 (9) Å	θ = 3.1–32.0°
α = 92.734 (5)°	µ = 0.09 mm^−^^1^
β = 100.608 (6)°	*T* = 123 K
γ = 108.989 (6)°	Block, colourless
*V* = 770.11 (10) Å^3^	0.19 × 0.08 × 0.05 mm

### (III) N-(5-Acetyl-2-ethynylphenyl)quinoline-2-carboxamide Data collection

**Table:**

Agilent SuperNova Single source at offset, Eos diffractometer	5173 independent reflections
Radiation source: micro-focus sealed X-ray tube, SuperNova (Mo) X-ray Source	3687 reflections with *I* > 2σ(*I*)
Mirror monochromator	*R*_int_ = 0.060
Detector resolution: 7.9851 pixels mm^-1^	θ_max_ = 32.3°, θ_min_ = 3.0°
ω scans	*h* = −10→10
Absorption correction: analytical [CrysAlis PRO (Rigaku OD, 2015), based on expressions derived by Clark & Reid (1995)]	*k* = −12→12
*T*_min_ = 0.987, *T*_max_ = 0.996	*l* = −20→20
20693 measured reflections	

### (III) N-(5-Acetyl-2-ethynylphenyl)quinoline-2-carboxamide Refinement

**Table:**

Refinement on *F*^2^	0 restraints
Least-squares matrix: full	Hydrogen site location: inferred from neighbouring sites
*R*[*F*^2^ > 2σ(*F*^2^)] = 0.056	H-atom parameters constrained
*wR*(*F*^2^) = 0.151	*w* = 1/[σ^2^(*F*_o_^2^) + (0.0577*P*)^2^ + 0.282*P*] where *P* = (*F*_o_^2^ + 2*F*_c_^2^)/3
*S* = 1.04	(Δ/σ)_max_ < 0.001
5173 reflections	Δρ_max_ = 0.42 e Å^−^^3^
218 parameters	Δρ_min_ = −0.26 e Å^−^^3^

### (III) N-(5-Acetyl-2-ethynylphenyl)quinoline-2-carboxamide Special details

**Table:**

Geometry. All esds (except the esd in the dihedral angle between two l.s. planes) are estimated using the full covariance matrix. The cell esds are taken into account individually in the estimation of esds in distances, angles and torsion angles; correlations between esds in cell parameters are only used when they are defined by crystal symmetry. An approximate (isotropic) treatment of cell esds is used for estimating esds involving l.s. planes.

### (III) N-(5-Acetyl-2-ethynylphenyl)quinoline-2-carboxamide Fractional atomic coordinates and isotropic or equivalent isotropic displacement parameters (Å^2^)

**Table:**

	*x*	*y*	*z*	*U*_iso_*/*U*_eq_	
O2	0.81853 (17)	0.81639 (13)	0.58241 (8)	0.0298 (3)	
O1	0.69300 (18)	0.80001 (15)	1.00770 (8)	0.0344 (3)	
N1	0.73126 (17)	0.52751 (15)	0.59954 (8)	0.0204 (2)	
H1	0.7132	0.4296	0.5678	0.025*	
N2	0.76917 (16)	0.45956 (15)	0.41516 (8)	0.0204 (2)	
C11	0.7866 (2)	0.66691 (18)	0.54929 (10)	0.0211 (3)	
C16	0.78375 (19)	0.41914 (18)	0.32020 (10)	0.0205 (3)	
C8	0.6340 (2)	0.49494 (18)	0.88964 (10)	0.0225 (3)	
H8	0.6117	0.4859	0.9536	0.027*	
C3	0.69438 (19)	0.65661 (18)	0.85563 (9)	0.0199 (3)	
C9	0.6242 (2)	0.20816 (18)	0.66981 (10)	0.0235 (3)	
C4	0.72565 (19)	0.67055 (17)	0.75912 (9)	0.0201 (3)	
H4	0.7637	0.7782	0.7364	0.024*	
C6	0.64210 (19)	0.36039 (17)	0.73190 (9)	0.0200 (3)	
C5	0.69995 (19)	0.52316 (17)	0.69665 (9)	0.0190 (3)	
C15	0.8349 (2)	0.54711 (19)	0.25516 (10)	0.0226 (3)	
C12	0.80629 (19)	0.62324 (17)	0.44536 (9)	0.0198 (3)	
C2	0.7259 (2)	0.81339 (19)	0.92427 (10)	0.0238 (3)	
C13	0.8613 (2)	0.75994 (18)	0.38704 (10)	0.0235 (3)	
H13	0.8881	0.8734	0.4126	0.028*	
C7	0.6074 (2)	0.34872 (18)	0.82831 (10)	0.0227 (3)	
H7	0.5661	0.2413	0.8511	0.027*	
C17	0.7457 (2)	0.24391 (19)	0.28605 (11)	0.0264 (3)	
H17	0.7138	0.1593	0.3282	0.032*	
C10	0.6224 (2)	0.0885 (2)	0.61968 (12)	0.0305 (3)	
H10	0.6210	−0.0063	0.5800	0.037*	
C14	0.8738 (2)	0.72010 (19)	0.29172 (10)	0.0249 (3)	
H14	0.9079	0.8069	0.2511	0.030*	
C20	0.8445 (2)	0.4964 (2)	0.15714 (10)	0.0283 (3)	
H20	0.8776	0.5790	0.1140	0.034*	
C18	0.7560 (2)	0.2000 (2)	0.19069 (12)	0.0309 (3)	
H18	0.7302	0.0851	0.1685	0.037*	
C19	0.8051 (2)	0.3267 (2)	0.12586 (11)	0.0315 (3)	
H19	0.8108	0.2945	0.0614	0.038*	
C1	0.8052 (3)	0.9875 (2)	0.89000 (11)	0.0326 (4)	
H1A	0.8080	1.0748	0.9392	0.049*	
H1B	0.7214	0.9926	0.8288	0.049*	
H1C	0.9369	1.0066	0.8801	0.049*	

### (III) N-(5-Acetyl-2-ethynylphenyl)quinoline-2-carboxamide Atomic displacement parameters (Å^2^)

**Table:**

	*U*^11^	*U*^22^	*U*^33^	*U*^12^	*U*^13^	*U*^23^
O2	0.0459 (7)	0.0218 (5)	0.0232 (5)	0.0113 (5)	0.0114 (5)	0.0015 (4)
O1	0.0440 (7)	0.0339 (6)	0.0211 (5)	0.0056 (5)	0.0126 (5)	−0.0035 (4)
N1	0.0256 (6)	0.0189 (5)	0.0162 (5)	0.0063 (4)	0.0057 (4)	0.0002 (4)
N2	0.0197 (5)	0.0215 (5)	0.0187 (5)	0.0054 (4)	0.0043 (4)	0.0001 (4)
C11	0.0238 (6)	0.0227 (6)	0.0167 (6)	0.0079 (5)	0.0042 (5)	0.0012 (5)
C16	0.0180 (6)	0.0240 (7)	0.0180 (6)	0.0060 (5)	0.0034 (5)	−0.0014 (5)
C8	0.0225 (6)	0.0279 (7)	0.0173 (6)	0.0077 (5)	0.0062 (5)	0.0034 (5)
C3	0.0189 (6)	0.0236 (6)	0.0161 (5)	0.0064 (5)	0.0032 (5)	−0.0011 (5)
C9	0.0234 (7)	0.0252 (7)	0.0224 (6)	0.0072 (5)	0.0071 (5)	0.0070 (5)
C4	0.0207 (6)	0.0211 (6)	0.0173 (6)	0.0055 (5)	0.0039 (5)	0.0012 (5)
C6	0.0195 (6)	0.0214 (6)	0.0186 (6)	0.0061 (5)	0.0041 (5)	0.0015 (5)
C5	0.0182 (6)	0.0231 (6)	0.0158 (5)	0.0067 (5)	0.0047 (4)	0.0026 (5)
C15	0.0192 (6)	0.0308 (7)	0.0164 (6)	0.0072 (5)	0.0031 (5)	0.0014 (5)
C12	0.0201 (6)	0.0217 (6)	0.0165 (5)	0.0058 (5)	0.0036 (5)	0.0006 (5)
C2	0.0241 (7)	0.0273 (7)	0.0184 (6)	0.0083 (6)	0.0027 (5)	−0.0022 (5)
C13	0.0280 (7)	0.0209 (6)	0.0196 (6)	0.0057 (5)	0.0045 (5)	0.0016 (5)
C7	0.0238 (7)	0.0225 (6)	0.0219 (6)	0.0066 (5)	0.0071 (5)	0.0044 (5)
C17	0.0250 (7)	0.0247 (7)	0.0278 (7)	0.0068 (6)	0.0061 (6)	−0.0023 (5)
C10	0.0358 (8)	0.0276 (7)	0.0272 (7)	0.0098 (6)	0.0065 (6)	0.0020 (6)
C14	0.0266 (7)	0.0266 (7)	0.0202 (6)	0.0059 (6)	0.0066 (5)	0.0063 (5)
C20	0.0256 (7)	0.0418 (9)	0.0177 (6)	0.0119 (6)	0.0048 (5)	0.0020 (6)
C18	0.0285 (8)	0.0317 (8)	0.0302 (8)	0.0103 (6)	0.0038 (6)	−0.0090 (6)
C19	0.0295 (8)	0.0453 (9)	0.0197 (6)	0.0152 (7)	0.0037 (6)	−0.0059 (6)
C1	0.0483 (10)	0.0241 (7)	0.0223 (7)	0.0107 (7)	0.0044 (6)	−0.0023 (6)

### (III) N-(5-Acetyl-2-ethynylphenyl)quinoline-2-carboxamide Geometric parameters (Å, º)

**Table:**

O2—C11	1.2279 (17)	C6—C7	1.4033 (18)
O1—C2	1.2231 (17)	C15—C14	1.411 (2)
N1—H1	0.8600	C15—C20	1.4195 (19)
N1—C11	1.3595 (18)	C12—C13	1.4130 (19)
N1—C5	1.4025 (16)	C2—C1	1.501 (2)
N2—C16	1.3704 (17)	C13—H13	0.9300
N2—C12	1.3197 (17)	C13—C14	1.3692 (19)
C11—C12	1.5082 (18)	C7—H7	0.9300
C16—C15	1.422 (2)	C17—H17	0.9300
C16—C17	1.421 (2)	C17—C18	1.372 (2)
C8—H8	0.9300	C10—H10	0.9300
C8—C3	1.397 (2)	C14—H14	0.9300
C8—C7	1.3799 (19)	C20—H20	0.9300
C3—C4	1.3979 (18)	C20—C19	1.367 (2)
C3—C2	1.4962 (19)	C18—H18	0.9300
C9—C6	1.4435 (19)	C18—C19	1.411 (2)
C9—C10	1.175 (2)	C19—H19	0.9300
C4—H4	0.9300	C1—H1A	0.9600
C4—C5	1.3979 (18)	C1—H1B	0.9600
C6—C5	1.4115 (18)	C1—H1C	0.9600
			
C11—N1—H1	115.9	O1—C2—C3	120.60 (13)
C11—N1—C5	128.21 (12)	O1—C2—C1	120.68 (13)
C5—N1—H1	115.9	C3—C2—C1	118.69 (12)
C12—N2—C16	117.64 (12)	C12—C13—H13	121.0
O2—C11—N1	125.11 (12)	C14—C13—C12	117.90 (13)
O2—C11—C12	121.14 (12)	C14—C13—H13	121.0
N1—C11—C12	113.75 (11)	C8—C7—C6	120.69 (13)
N2—C16—C15	121.91 (12)	C8—C7—H7	119.7
N2—C16—C17	118.75 (13)	C6—C7—H7	119.7
C17—C16—C15	119.33 (12)	C16—C17—H17	120.1
C3—C8—H8	120.0	C18—C17—C16	119.78 (14)
C7—C8—H8	120.0	C18—C17—H17	120.1
C7—C8—C3	119.98 (12)	C9—C10—H10	180.0
C8—C3—C4	120.17 (12)	C15—C14—H14	120.0
C8—C3—C2	118.92 (12)	C13—C14—C15	119.92 (13)
C4—C3—C2	120.92 (12)	C13—C14—H14	120.0
C10—C9—C6	175.76 (16)	C15—C20—H20	119.8
C3—C4—H4	119.9	C19—C20—C15	120.33 (15)
C3—C4—C5	120.21 (12)	C19—C20—H20	119.8
C5—C4—H4	119.9	C17—C18—H18	119.5
C5—C6—C9	119.94 (12)	C17—C18—C19	120.92 (14)
C7—C6—C9	120.52 (12)	C19—C18—H18	119.5
C7—C6—C5	119.51 (12)	C20—C19—C18	120.49 (14)
N1—C5—C6	117.23 (12)	C20—C19—H19	119.8
C4—C5—N1	123.36 (12)	C18—C19—H19	119.8
C4—C5—C6	119.41 (12)	C2—C1—H1A	109.5
C14—C15—C16	117.90 (12)	C2—C1—H1B	109.5
C14—C15—C20	122.96 (14)	C2—C1—H1C	109.5
C20—C15—C16	119.14 (13)	H1A—C1—H1B	109.5
N2—C12—C11	117.50 (12)	H1A—C1—H1C	109.5
N2—C12—C13	124.71 (12)	H1B—C1—H1C	109.5
C13—C12—C11	117.78 (12)		
			
O2—C11—C12—N2	−179.91 (13)	C9—C6—C5—C4	−177.04 (12)
O2—C11—C12—C13	−0.5 (2)	C9—C6—C7—C8	176.73 (13)
N1—C11—C12—N2	−0.07 (18)	C4—C3—C2—O1	177.96 (13)
N1—C11—C12—C13	179.34 (12)	C4—C3—C2—C1	−4.1 (2)
N2—C16—C15—C14	0.9 (2)	C5—N1—C11—O2	−0.2 (2)
N2—C16—C15—C20	−179.00 (12)	C5—N1—C11—C12	180.00 (12)
N2—C16—C17—C18	178.87 (13)	C5—C6—C7—C8	−1.6 (2)
N2—C12—C13—C14	1.2 (2)	C15—C16—C17—C18	−0.9 (2)
C11—N1—C5—C4	−1.0 (2)	C15—C20—C19—C18	−0.5 (2)
C11—N1—C5—C6	179.84 (13)	C12—N2—C16—C15	−0.63 (19)
C11—C12—C13—C14	−178.19 (12)	C12—N2—C16—C17	179.63 (12)
C16—N2—C12—C11	178.94 (11)	C12—C13—C14—C15	−0.8 (2)
C16—N2—C12—C13	−0.4 (2)	C2—C3—C4—C5	178.53 (12)
C16—C15—C14—C13	−0.1 (2)	C7—C8—C3—C4	0.8 (2)
C16—C15—C20—C19	−0.1 (2)	C7—C8—C3—C2	−178.82 (13)
C16—C17—C18—C19	0.4 (2)	C7—C6—C5—N1	−179.53 (12)
C8—C3—C4—C5	−1.1 (2)	C7—C6—C5—C4	1.28 (19)
C8—C3—C2—O1	−2.4 (2)	C17—C16—C15—C14	−179.36 (13)
C8—C3—C2—C1	175.51 (13)	C17—C16—C15—C20	0.7 (2)
C3—C8—C7—C6	0.5 (2)	C17—C18—C19—C20	0.3 (2)
C3—C4—C5—N1	−179.10 (12)	C14—C15—C20—C19	−179.97 (14)
C3—C4—C5—C6	0.04 (19)	C20—C15—C14—C13	179.78 (13)
C9—C6—C5—N1	2.14 (18)		

### (III) N-(5-Acetyl-2-ethynylphenyl)quinoline-2-carboxamide Hydrogen-bond geometry (Å, º)

**Table:**

*D*—H···*A*	*D*—H	H···*A*	*D*···*A*	*D*—H···*A*
N1—H1···N2	0.86	2.23	2.666 (2)	111
C10—H10···O2^i^	0.93	2.36	3.103 (2)	136

Symmetry code: (i) *x*, *y*−1, *z*.
